# Medical misinformation in Lebanese media: A qualitative study of Stakeholders’ perspectives and policy gaps

**DOI:** 10.1371/journal.pgph.0006277

**Published:** 2026-04-08

**Authors:** Mohamad Sadek Zoghbi, Adnan Fatfat, Diala Jazra, Elio R. Bitar, Karine Eid, Razane Wehbe, Sarah Helmy, Yeva Fakih, Nisrine Makarem, Salim Adib

**Affiliations:** 1 Faculty of Medicine, American University of Beirut, Beirut, Lebanon; 2 Department of Family Medicine, American University of Beirut, Beirut, Lebanon; 3 Department of Epidemiology and Population Health, American University of Beirut, Beirut, Lebanon; PLOS: Public Library of Science, UNITED STATES OF AMERICA

## Abstract

The spread of medical misinformation through conventional and online communication poses a public health risk, especially in countries like Lebanon, where the healthcare system is fragile. Its dangers were highlighted during the COVID-19 pandemic and other health emergencies, showing how inaccurate or misleading information can alter public health behavior. Despite growing awareness about the problem, no study in Lebanon has explored health misinformation from the perspective of key healthcare stakeholders. The aim of this study was to investigate the views of key stakeholders on the causes and implications of health-related misinformation in Lebanese media, and to provide recommendations to combat it. We conducted a qualitative study using semi-structured, in-depth interviews with nine elite interviewees from the media, health sector, government, professional syndicates, and legal system. Interviews were conducted between March 17 and 25, 2025 and thematically analyzed using an inductive-deductive approach. Data saturation was reached at the eighth interview. Trustworthiness was ascertained through triangulated coding, reflexivity, and adherence to established qualitative rigor criteria. Factors contributing to the spread of health misinformation included low health literacy, poverty, political pressure on the media, and a lack of regulations on digital platforms. WhatsApp, Facebook, and Instagram were identified as major channels for rapid dissemination. Although some Lebanese media outlets practiced voluntary fact-checking and consulted experts, these efforts remained intermittent and unenforced. Government responses were described as reactive, relying more on public credibility than legislation. Existing health legislation was seen as outdated, weakly enforced, and disconnected from digital realities. The COVID-19 pandemic exposed these vulnerabilities and highlighted the need for coordinated broadcasting approach and stronger expert oversight across media platforms. Medical misinformation in Lebanon is driven by structural weaknesses across media, health, and legal systems. Addressing it requires coordinated institutional action, improved health literacy, stronger collaboration between media and health professionals, and updated regulations suited to the digital era.

## Introduction

In an era of rapid spread of information, medical myths can lead to disastrous consequences. A striking example was provided on a well-recognized Lebanese TV channel, where a television report declared the beta blocker, Concor, can have fatal consequences on the cerebral circulation of blood and oxygen [1]. This report prompted widespread concern, with many patients contacting their physicians to ask whether they should discontinue the medication. Other remarkable incidents include widespread false information during the COVID-19 pandemic, such as audio voice notes on WhatsApp spreading conspiracy theories about the virus, creating mass panic and reduced adherence to public guidelines [2]. Similarly, a regional study reported conspiracy beliefs propagated via social media associated with hesitation to take the vaccine, such as vaccines implanting microchips (27.7%) or causing infertility (23.4%) [3]. These incidents demonstrate how false information in the mass media can instantaneously influence the behavior of the public and lead to life-threatening results. Likewise, this issue highlights the need to study the role of public media in health communication.

False health-related content typically takes two forms: misinformation and disinformation. Misinformation refers to false, inaccurate, or misleading health information published without the intention of misleading due to misunderstanding, misinterpretation, or outdated information [4]. On the other hand, disinformation involves the deliberate spread of false or misleading information about health with the aim of misleading individuals, typically for political, financial, or selfish purposes [5]. World Health Organization (WHO) has identified health misinformation as a serious threat to global health and security, with some reports blaming it for lowered vaccine trust and increased consumption of untested remedies [6]. Lack of control over health-related content has had dire public health consequences, especially during the COVID-19 pandemic. The dissemination of misinformation has also had negative outcomes, such as vaccine hesitancy, fear, and ultimately, avoidable outcome [7,8]. These consequences are magnified by the huge structure of today’s media digital platforms

Social media use has grown exponentially, with an estimated 5 billion users worldwide [9]. Social media platforms like Facebook, Instagram, TikTok, and YouTube have become significant sources of health information, at times ranking above traditional news sources in perceived credibility [10]. This phenomenon places users in the challenge of identifying credible and non-credible sources and making it harder for them to identify trustworthy information [11]. As a result, misinformation, unfounded information, and false claims frequently coexist with evidence-based medical advice, undermining clinical outcomes, public trust in medical institutions, and frustrating policymakers and practitioners [12]. Beyond the COVID-19 pandemic, misinformation is also common in topics such as opioids, noncommunicable diseases, and smoking products. Studies have shown that online discussions about drug use and smoking are particularly afflicted with high levels of misinformation, which is compounded by algorithms that prioritize high engagement but often erroneous content over factually accurate information [13].

An understanding of how countries are addressing health misinformation is extremely policy relevant. In Lebanon, the spread of misinformation is multi-dimensional, driven by political instability, decentralized healthcare, and low public trust in official institutions. Media thus became a main and reliable source of information among the Lebanese population. Makhoul et al. found that the strong social cohesion of Lebanon facilitated rapid spreading of correct as well as incorrect information [14]. Lacking government public communication, the public becomes over-reliant on unofficial channels such as social media and local leaders, making decisions based more on social convention and economic hardship than on science. In contrast, the United Arab Emirates was graced with centralized leadership, high levels of institutional trust, and consolidated health messaging. Alam explained how collaboration between health authorities, police, and media under a National Response Framework made open, timely communication possible [15,16]. Misinformation was closely monitored and punished, and public trust in official sources strengthened compliance with health guidelines [16].

This study examines Lebanon’s active policy responses to health misinformation, the challenges facing media and health institutions, and proposes informed recommendations to policymakers and media stakeholders to counteract the dissemination of health misinformation and improve health communication. Although focused on Lebanon, the findings have implications for low- and middle-income countries (LMICs) with similarly fragile media ecosystems. Our study focuses on health misinformation in media, while also acknowledging the role of disinformation.

## Methods

### Study design

We conducted a qualitative study using semi-structured interviews to review the role and experiences of stakeholders in addressing health misinformation in Lebanon. We chose this design because it allows for in-depth exploration of social, cultural, and institutional insights that quantitative methods might overlook, which is essential for understanding policy gaps and stakeholder roles in a context like Lebanon’s fragmented media landscape.

### Recruitment

We identified participants after rigorous group discussions between the medical students and their tutors in the public health department, with the aim of identifying the key informants from large organizations such as the Ministry of Public Health and the media. It was important to identify the elite stakeholders since they are crucial decision-makers in the policies of media organizations, among others. Broader public views (such as patients, grassroots community members, nurses, or mid-level media staff) were beyond this study’s scope. We used snowball sampling to access hard-to-reach experts, where initial participants referred others, ensuring diverse perspectives. We selected participants based on their potential expertise in healthcare, media, law, or policy related to health communication in Lebanon. This expertise included mainly senior roles, such as directors, ministers, physicians, advisors, and lawyers. Policy-makers included former/current ministers and advisors involved in health regulation. We included participants that holds a senior role in their relevant sector and were ready to discuss health misinformation. We excluded participants who lack direct involvement in health policy/media or were unable to provide informed consent. We reached out to the informants directly through phone calls or emails. We provided them with information about the research project’s topic, explained the necessity of conducting interviews with them based on their positions or roles, and obtained their verbal consent before scheduling interviews. Informants were contacted until data saturation was reached, defined as the point at which no new major themes emerged during analysis.

### Data collection

We conducted the interviews between 17 and 25 March, 2025. Semi-structured interviews were either in-person or conducted via Zoom/Webex, contingent upon participant availability. The research team developed a study-relevant interview guide based on a review of relevant literature and refined through team discussions The guide included core questions for all participants along with additional role-specific questions. Topics covered were public health literacy, misinformation problems, and measures that might improve correct health information dissemination. All interviews were recorded (or recorded via Zoom) after obtaining participant consent and subsequently transcribed verbatim. Notes were taken immediately after each interview to capture contextual details.

### Interview questions

Interview questions explored a variety of stakeholder experience domains, such as:

- Participant role in health misinformation fighting
- Source credibility determining criteria before distributing health content (such as algorithmic verification, mandatory fact-checking for health content, and user education on credible sources)
- Techniques to verify accuracy of medical information
- Roles assumed during public health crises, such as COVID-19
- Perceptions about regulating digital media and misinformation’s behavioral impact

Other questions touched on sociodemographic determinants of exposure to misinformation—especially among marginalized populations—and health message tailoring strategies. Coordination of stakeholders (e.g., health workers, government, media), the “fake news” phenomenon, television and social media use in public health, and the main sources of misinformation in Lebanese media were also discussed by participants. The guide concluded with questions regarding potential awareness campaigns and the engagement of healthcare providers in referring their patients to trusted medical information. The full question guide can be found in S1 File.

### Data analysis

Interviews were done in Arabic, which was the preferred language of the participants. They were transcribed word for word in Arabic. Analysis was done in Arabic to allow for the nuances to be captured. Quotes were then translated to English as key quotes to determine their meaning. It was checked for accuracy of meaning by two different researchers independently. In the inductive deductive approach, coding was done on the basis of the theme arising and on existing concepts [17]. Firstly, all tapes were initially analyzed separately and privately by two team members for a familiarization stage. Prior to proper coding, there were repeated readings on the tapes. Second, uncoded ‘initial codes’ were introduced using an inductive data pattern, further deductively matching literature (e.g., highlighting issues on gap areas in terms of policies). Initial coding conducted through team discussions. Initial coding manually used Microsoft Word for coding transcripts and Microsoft Excel to codify team discussions through spreadsheets. This activity involved synchronized work among two team members. To ensure a reliable coding format, the first three transcripts comprised another 33% of the sample set, which were separately coded among two team members. Approximately an 85% level of coding agreement was estimated through a mutual comparison in Microsoft Excel. Disputed coding areas were resolved privately in team sessions, which were later moderated among a team member arbitrator. The codes were constantly improved, combined, and sub-categorized and further categorized according to viewpoints that came up. The quotes that were significant in relation to themes were pulled out. The saturation level was gauged by finding that there were no new themes left after conducting the 8th interview. At the end stage, there were no new findings regarding codes that ensured that there was theoretical saturation [18]. Ensuring the trustworthiness of the findings involved applying the criteria suggested by Lincoln and Guba [19]. These criteria can be summarized as follows: Ensuring Credibility involved prolonging the contact between the researcher and the data and carrying out member checking (presenting findings to participants for confirmation). Ensuring Transferability required the use of a thick description to identify the context. Ensuring Dependability involved keeping an audit trail to record coding decisions. Ensuring Confirmability required exercising reflexivity to consider personal biases, for example, a biomedical and media background. For example, the medical expertise in the team aided in the nuanced unpacking of medical misinformation while requiring careful bracketing in order not to be skewed towards healthcare-focused themes. The exercise aided in the reduction of preconceptions, like assuming there was always a nefarious intention by the media. The coding framework is illustrated with examples in the codebook presented as S1 Table.

### Ethics statement

This research is part of the Social & Preventive Medicine course, offered for fourth-year medical students. It is qualitative research that involves anonymous interviews among the adult stakeholders, including public policies, processes, as well as practices that are currently performed in Lebanon. There were no personal or private data gathered about medical topics. Thus, the research did not need a complete IRB protocol review as exempted by the American University of Beirut, as indicated in the course material which can be found on the attachments as S2 File [20]. All participants were informed about the purposes, methods, potential risk, as well as potential benefits associated with the research.

## Results

A total of 14 individuals were approached for the study, 5 people did not answer, and 9 individuals agreed to participate. Declines were due to scheduling conflicts or non-responses. Of the interviews, 8 were conducted over Zoom and 1 was conducted face to face. Enlisted below are brief descriptions of the interviewees contacted, their identities being kept anonymous:

Interviewee 1: Director of the Health section on a popular Lebanese TV channel
Interviewee 2: Director of the Health section on a popular Lebanese News outlet (social media and Newspaper)
Interviewee 3: Physician at a big university hospital in Lebanon.
Interviewee 4: Head of the Lebanese Order of Physicians
Interviewee 5: Former Minister of Health and surgeon at a big university hospital in Lebanon.
Interviewee 6: Director of the direct E-health program in the ministry of Public Health
Interviewee 7: Current Advisor for the Ministry of Public Health
Interviewee 8: Doctor, Former Member in the parliament and Lebanese Order of Physicians
Interviewee 9: Lawyer and member of the IRB legal counsel at a big university hospital in Lebanon.

Results of the content analysis were categorized to the following themes as following:

### 1.  Societal and information environment

#### a. Stakeholders’ perceptions of public health literacy.

Several participants noted that limited science education and health literacy levels are key drivers of misinformation spread in Lebanon. Interviewee 2, a media professional, elaborated:

*“There’s a lack of awareness and education around scientific and health issues in Lebanon. People are used to talking about politics and social issues, but health and science journalism are not well developed in this country.”*

To counteract this, Interviewee 2 referred to efforts to employ less complex language, infographics, and material that disadvantaged groups, particularly those with less formal education, can access. Low literacy and educational inequalities greatly influence the way individuals interact with health information. In poorly educated communities, people can be unable to critically assess the validity of claims by doctors, particularly when written in technical jargon. As one healthcare provider put it:

*“Socioeconomic status and education level and literacy based on the population may cause the spread of misinformation. Illiterate individuals are quick to believe misinformation.” – Interviewee 6*

Interviewee 1, also from the media sector, talked about the challenge of simplifying without sacrificing accuracy:

*“We, as journalists and directors of news channels, take many courses, do a lot of research and gain a lot of work experience before we master the art of simplification. We also know that not everyone is at the same level, especially in Lebanon, but our message, no matter how hard its level to understand, should be directed to the population in general.”*

Interviewee 1 also cautioned that Lebanese media is influenced by various political and economic interests, and thus people need to critically assess both content and source credibility:

*“In Lebanon particularly, there is an enormous political and economic sway over media reports, less so if the information is about health, which is a positive thing I imagine. There is almost always a secondary hidden gain.”*

Medical misinformation was portrayed as a cultural issue by Interviewee 3:

*“They have no sense of responsibility in verifying information before they pass it along.”*

Perception through culture and religion also affects how a person receives and believes in health messages. Misinformation that is thought to agree with preexisting worldviews is easier to accept even in the absence of evidence supporting it.

*“People are likely to believe information that is consistent with their religious or cultural beliefs. If health information is consistent with what they’ve learned or what their community holds to be true, they’re more likely to believe it without questioning its validity.” – Interviewee 6*

Language barriers and poor translation enable distortion of medical information, especially when scientific content of significance is distorted.

*“Occasionally poorly translated information will be misread and relayed incorrectly. Important medical information could be lost or become distorted while translating in certain cases.” – Interviewee 6*

Several participants discussed how socioeconomic disparities, access to digital technologies, and trust in institutions shape how different groups receive and process health information.

Participants also linked the country’s ongoing economic crisis to increased exposure to misinformation. As more individuals shun costly medical consultations, they resort more to online information, typically from unregulated or unverified sources:

*“The ongoing economic crisis has rendered healthcare less accessible, pushing people to the internet for explanations. Many people cannot afford a hospital visit, so they resort to the internet. subjecting them to misinformation.” — Interviewee 4*

This theme underscored a widespread vulnerability to health misinformation among the Lebanese public, founded on systemic underinvestment in science education, polarized media, and economic hardship

#### b. Media outlets’ role.

Interviewee 1, Director of the Health Section at MTV, emphasized the challenge of misinformation in an environment where “anyone can make an Instagram page and start giving opinions and advice to the public.” Social media platforms such as Instagram, Twitter, and Facebook have amplified unverified content, often driven by sensationalism. Mainstream media, Interviewee 1 clarified, may inadvertently aggravate the problem when pushed to respond quickly to viral news:

*“One of the greatest challenges is the speed at which misinformation gets around, and it tends to get around quicker than individuals who are trying to debunk it. People tend to believe what they hear first, even when it’s not true.”*

Yet, several media organizations are actively attempting to stop misinformation. Interviewee 1 discussed how her organization is in an active collaborative relationship with Clemenceau Medical Center, which is affiliated with Johns Hopkins, to medically screen content. MTV also has a special segment that works to debunk health myths and draw attention to urgent public health issues.

*“We always refer to medical professionals to fact-check health claims before we go on air. We also have a specific TV segment for dispelling misinformation and creating awareness on important health issues.”*

Interviewee 2, Director of Al-Nahar’s Health Section, also emphasized a firm commitment to reputable sourcing. Their newsroom relies on institutions such as Mayo Clinic, WHO, and Harvard Medical School. They even have a “fake news” section, which was very active during the COVID-19 pandemic. However, Interviewee 2 admitted that these practices are not followed by all Lebanese media, since some prioritize engagement over accuracy:

*“Part of the media focuses on clicks rather than accuracy, and this boosts the spread of misinformation.”*

Al-Nahar’s editorial policy is to check with several medical experts and to delay publication where necessary to ensure credibility:

*“Even if the topic is not local or breaking news, we always refer back to established international agencies. We prioritize solid medical information.”*

Both interviewees highlighted the media’s role in debunking misinformation, given their large audiences.

*“We attempt to actively search for misinformation on social media, fact-check with physicians, and then give corrections on our news website. Accountability remains a huge issue.” – Interviewer 1*

Interviewee 1 clarified, however, that the intention was educational

*“Our segment attempted to introduce potential side effects to viewers who may lack access to immediate medical consultation. It was never our aim to panic or lead patients to stop medication without a doctor’s recommendation.”*

Interviewee 1 also highlighted the rigorous preparation required for responsible health journalism, describing how her team has continuous training, expert consultations, and multi-level editorial check processes:

*“There’s no formal system of accountability—it’s up to every individual to do the work in good conscience.”*

### 2. Institutional and policy context

#### a. Health-care sector involvement.

Interviewee 3, a physician at AUBMC and a health-focused Instagram content creator, emphasized the dual responsibility of being a clinician and a public influencer:

*“I see it as a big responsibility to share accurate health information with the public. My goal is to empower patients with knowledge.”*

Interviewee 3 noted that their Instagram account has become a platform to correct myths frequently raised by patients in clinical settings:

*“They keep messaging me in DMs about healthcare myths that aren’t true, and it’s a duty for me to debunk and correct them.”*

Interviewee 3 emphasized the need for greater involvement of medical practitioners in fighting misinformation:

*“It’s our duty, as people with medical knowledge, to give proper health information to others who have less access to healthcare.”*

Interviewee 4, another clinician, also referred to the double effect of online research. Empowered patients might make consultations more effective, but alongside this is the risk of misinformation leading to lethal self-diagnosis:

*“When patients do appropriate online research prior to appointments, it does help. But the reverse—self-diagnosis using fallacious content—can be dangerous.”*

*“Certain claims indicate that surgeries can be prevented with alternative treatments, which is very misleading.” – Interviewee 4*

Interviewee 3 too had concerns regarding the indirect role of the drug industry in disseminating misinformation, specifically through fear marketing:

*“They are afraid they won’t be able to conceive, but the test doesn’t mean that. A lot of times, it is due to companies wanting to sell IVF or other drugs.”*

Interviewee 3 emphasized the necessity of increasing evidence-based material and not just censoring false information:

*“We must continue to drive home rock-solid, fact-based messages—time and time again. If one’s intention is to educate—not just to gain followers—they owe it to others to report accurately.”*

Interviewee 3 noted that personal stories receive much more traction than fact-based information:

*“One of my initial goals is to share scientific knowledge, but it doesn’t always work well on social media. I like to employ more stories, which is one of the most powerful ways to engage people.”*

Interviewee 3 felt that websites today have more influence than the traditional methods:

*“Information is literally at people’s fingertips—it’s in their hands, on their phones. We need to ask ourselves—how do individuals want to access their information? In a pamphlet? Or from a trusted physician on Instagram?”*

To many, she believes, the answer is clear.

#### b. Government interference.

Interviewees with direct exposure to the government explain how the ministry functions in this complex context, particularly in times of crisis.

Interviewee 5, who is a professional surgeon and ex-Minister of Public Health (2021–2025), sketched the ministry’s evolving reaction to the COVID-19 crisis. Their own tweeting during this period turned their account to “almost the go-to place to get information about COVID in Lebanon,” which they experienced “more or less a very positive experience” in calming public fears. Good crisis communication, they emphasized, is a necessary condition:

*“The last thing you want is panic.”*

Interviewee 5 compared media to a clean tool:

*“Just like a car can be used to save lives or injure people, it is up to the purpose you use it for.”*

Expanding on their earlier hospital leadership during the COVID-19 pandemic, Interviewee 5 emphasized the importance of “controlling the narrative,” particularly during crises. As one of the main players in the national wartime disruption response, they led the creation of the Public Health Emergency Center, grounding its daily releases on the COVID-era hospital news.

*“We became the go-to for anything in public health. The difficult part is not becoming a go-to its remaining one.”*

Interviewee 5 applies rigorous criteria before sharing health information from a scientific communication perspective: journal credibility, peer-reviewed status, methodological clarity, and clinical reasoning compatibility.

*“If it is scientifically sound, you can agree. If not, you quote it but criticize it.”*

Although they knew that “medicine is not an exact science,” they forbade taking any opinion as dogma: “In science, nothing is dogma.”. Rather than inclining towards hard censorship, Interviewee 5 suggested investing in long-term public awareness:

*“The more you invest in health literacy and public awareness, the harder it is for misinformation to take hold.” “This is why it is so important that the Ministry of Public Health position itself as a trusted point of reference.” You must speak up, they emphasized. “If you don’t speak up, you’ve let the people with misinformation win. People are more drawn to sensational stories than grave medical news.”*

Health literacy was a key concern, with Interviewee 5 describing how the less literate is more prone to conspiracy theories—microchips in vaccines.

*“Health literacy determines how vulnerable you are, you’re either immune or vulnerable.”*

To reach diverse groups, they stressed the necessity to target older populations via television and young people via social media.

*“If you want an all-out campaign, everybody has to be accessed.”*

In getting to the sources of misinformation, Interviews 5 reported influencers’ variable literacy:

*“If influencers are health literate, it reflects in their content. If they’re not, their followers are misinformed.” Instead of trying to reach everyone, they advocated for reaching influential content creators. “We need to leave the hospital and reach communities,” as one example.*

In terms of regulation, Interviewee 5 indicated that unhealthy media content is controlled by legislation under the Ministry of Information, not Health.

*“The law in general states that media cannot harm members of the public, but this is not well defined and hasn’t been updated. I would not say that this is a well-controlled area.”*

Interviewee 5 informed that Lebanon’s national health strategy (2023) includes communication and misinformation as top priorities.

*“Public health experts are working on its implementation. They felt they were raising awareness, but actually, they normalized suicide”*

Interviewee 5 said. To address this, therefore, MOPH collaborated with the Ministry of Information to inform journalists.

*“We had targeted education sessions. I believe it is the mandate of the Ministry of Health to educate—not only the masses but also influencers like journalists.”*

Interviewee 7, previously (2020–2022) and currently the advisor to the MOPH, cited a functional issue: there are no laws you can enforce against misinformation.

*“There are no specific regulations per se. What we do is monitor the media and respond with clarifying statements.”*

The ministry’s authority usually comes from popular faith as opposed to legislation.

*“If MOPH releases are out there, they shut down any discussion of misinformation because they override unconfirmed sources,”* Interviewee 7 articulated.

Interviewee 6, Director of the E-Health Program at the Ministry, addressed the ministry’s impact on digital health communication. Her unit manages the official website, social media, and public health messaging with the Health Promotion Department.

*“We work together to provide accurate and accessible health information,”* she said.

For the prevention of misinformation, the E-Health team includes medical doctors who vet content before it is published. The ministry also works with the World Health Organization (WHO) for harmonization of messaging to international standards and works in close coordination with the National News Agency and Ministry of Information towards upholding credibility on media outlets.

*“We are advised by a professional health team before publishing anything. We also get coordinated with the WHO and with national media groups so that we have uniform, accurate communication”* Interviewee 6 revealed.

#### c. Current legislation and regulations.

Legal experts and doctors agree that there is no distinct, enforceable legal model to rectify medical misinformation, especially on the Internet. Interviewee 9, a lawyer and legal counsel to an IRB at a major medical university, highlighted the uncertainty of prosecution. Misinformation is common, but prosecution is rare and requires demonstrable harm—a stringent criterion that few cases meet.

*“Prosecution only if harm is proven. There are no specific laws regarding medical misinformation—it falls under the general media law and code of conduct for journalists.” – Interviewee 9*

*“Legal advice cannot prosecute by itself. There must be a claim of injury by a person, or by physicians through the Order of Physicians, which gains more weight and credence.” – Interviewee 9*

*“Health misinformation is not easy to prosecute. Physicians are most likely to detect it, but the enforcement must be carried out by the MoPH, the institutions of justice, and the Order of Physicians. There must be crystal-clear laws. it’s collective responsibility.” – Interviewee 4*

*“Internet law remains vague. We don’t rely on it and fall back on ancient 1943 media statutes to extrapolate from.” – Interviewee 9*

*“Anyone can sue, but it takes a great deal of time and money. A case that resolves in less than five years is optimistic.” – Interviewee 9*

The Lebanese press has weak enforcement and poor accountability. While some papers self-correct upon receiving expert advice, no legal repercussions attend the spread of false information.

*“If a media outlet posts incorrect information, there is no real penalty other than public outcry. In a country like Lebanon, it’s every person’s work ethic, moral compass, and personal code. There is no one behind you but you.” – Interviewee 1*

*“If not injuring, the goal should be to stop it—not sue. Syndicates like the Order of Physicians are our best defense.” – Interviewee 9*

This was supported by Interviewee 4, president of the Lebanese Order of Physicians. The order works hand in hand with the Ministry of Public Health in developing ethical and legal standards maintaining confidentiality as well as truthfulness of information.

*“We want to come up with legislation that protects medical information. There’s a need for ethics committees to enforce standards. There’s wide scope to amend these tasks.” – Interviewee 4*

*“The Order must be in a position to undertake formal investigations against public health claims, especially those that lack scientific evidence. The syndicate leaders are entitled to a role to play.” – Interviewee 4*

Interviewee 8, a former regulator, lamented that there is no institution that can address misinformation on a systemic basis.

*“Laws need accurate medical information, but there is no institution to supervise or counter misinformation. The Central Inspection Authority once functioned—but no longer does.” – Interviewee 8*

Political apathy, poor regulation, and lack of cooperation between sectors add to the weakening of the ability to address misinformation.

*“Reaching diverse communities requires political will and intersectoral collaboration.” – Interviewee 8*

Beyond structural weaknesses, actual misinformation is particularly dangerous. Misinformation is sometimes used intentionally to undermine public faith, influence attitudes, or further politics.

*“Deceptive information is relayed with the intention of influencing public opinion, destabilizing the government, or for political purposes.” – Interviewee 6*

Commercial interests can also fuel misinformation, with companies promoting inaccurate claims to market products.

*“Medical misinformation disseminates for commercial purposes, as companies distribute false information to the market and sell their products.” – Interviewee 8*

Journalists are faced with numerous constraints in rebuffing misinformation They are often asked to verify facts without recourse to up-to-date data—such as cancer statistics that have not been updated since 2015. Further, budgetary constraints limit the feasibility of investigative health reporting in the long term.

*“Politics and economics always take priority here. For serious topics like the health effects of war or pollution, we’d need long-term research and collaboration with labs and researchers. Right now, most initiatives are individual.” – Interviewee 2*

The global nature of online platforms complicates regulation, requiring multilateral coordination to establish meaningful enforcement mechanisms.

*“We can pass pre-authorization legislation on some content, but enforcement is restricted without international cooperation.” – Interviewee 4*

Finally, even when false information has been found, it is difficult to step in without established legal structures and multi-agency collaboration. Health professionals are likely to spot false claims first, but enforcement must be concerted.

### 3. Lessons learned: What have we learned from the COVID-19 infodemic?

*“Early COVID, we knew very little about the virus, and this allowed misinformation to circulate. Some even claimed the virus never existed. Then, more recently, there were vaccine misinformation disseminators emphasizing reports of severe side effects and death.” – Interviewee 7*

The Ministry of Public Health (MoPH) subsequently picked up universally accepted practices, introducing only FDA- or WHO-approved vaccines and taking national policy in line with WHO guidelines.

*“By embracing WHO guidelines, we provided consistent health recommendations and dispelled rumors.– Interviewee 7*

As a response to misinformation combat, MoPH undertook several targeted strategies:

- Transparent daily COVID-19 briefings to redirect attention towards legitimate sources.
- Health specialist media engagement to prevent unqualified actors from dominating the discussion.
- Media monitoring units to monitor and correct misinformation.
- The “Fact Check” website, with the Ministry of Information’s collaboration, presented a single webpage to verify claims in circulation.
- A COVID-19 chatbot presented rapid, evidence-based responses to the most frequent questions the public had on their minds.

Social media monitoring, particularly Facebook, actively enabled the Ministry to openly counter viral misinformation by publishing false claims and presenting them alongside factual corrections.

*“Fact Check served as a public window through which people could verify COVID-related info. It was meant to address public worries as well as dispel misinformation.” – Interviewee 7*

*“The chatbot provided quick, accurate responses to routine COVID-related questions, helping the public navigate uncertainty.” – Interviewee 6*

*“We monitored Facebook for misinformation and responded back with accurate health guidance. The approach led to observed changes in public behavior.” – Interviewee 6*

*“We interviewed doctors daily, fact-checked rigidly, but sometimes guidelines changed overnight, making previous coverage outdated.” – Interviewee 1*

*“Medical misinformation is riskier than political or economic misinformation because it directly affects people’s health.” – Interviewee 2*

*“There have been no large lawsuits regarding COVID-19 misinformation. It is nearly impossible to win such cases, particularly involving hospitals or physicians. Their legal system in Lebanon is nearly impenetrable.” – Interviewee 9*

The health workers also played a direct role in public education and media engagement.

*“I responded to media and public inquiries, ensuring that accurate information was accessible to counter false narratives.” – Interviewee 8*

### 4. Prospective plans

Interviewee 2, who is a journalist, highlighted how reliable media coverage of the COVID-19 pandemic relied on consulting specialists:

*“When a topic produces differing opinions, we summon different doctors or specialists to provide different opinions, always presenting the information as it is, without manipulation.” – Interviewee 2*

*“Other media outlets care more about clicks than accuracy, even at the cost of good information. And health reporting in Lebanon is underdeveloped discussions are more about politics than medicine or science.” – Interviewee 2*

Interviewee 4, a public health officer, called for greater institutional involvement in public health education:

*“Despite Lebanon being very connected digitally, access to trustworthy information remains an issue. There must be an active educational role, led by the MoPH and Ministry of Information. It has to be a concerted effort to direct communities to trustworthy sources and close health literacy gaps.”*

*“Real development requires political will in acting in the interest of public health over sectarian politics. Building alliances to safeguard scientific integrity in public discourse can lead to opening the door to a more informed society.” – Interviewee 8*

*“The Ministry of Public Health, Ministry of Information, Higher Council of Health, and syndicates must work together. We must address underlying issues, not issues on the surface. Organizations such as the World Bank are able to contribute towards funding these reforms.” – Interviewee 4*

*“We need direct communication between scientific institutions and the public, tailored to people of all ages and backgrounds.” – Interviewee 4*

*“These should be preventive and intersectoral. Hospitals, NGOs, schools, and clinics must cooperate under government control. We must teach people how to identify credible sources—and outline how misinformation can be more destructive than helpful.” – Interviewee 4*

## Discussion

This research’s findings chronicled the complex nature of medical misinformation in Lebanon, entrenched in challenges in media, health, and government institutions. Interviews with stakeholders across various sectors showed that there were inherent structural gaps in the regulation of health information and the effectiveness of public health communication interventions. This has made it difficult for Lebanon to mitigate the growing persistence of misinformation during times of crises.

### Media and regulation

Media organizations, especially those with broad viewership and public trust, are also important in shaping public health beliefs and attitudes. In Lebanon, several research participants pointed to the two-fold nature of the media as a spreader of misconceptions as well as a platform capable of repeating correct health messages. Lebanese media practitioners are aware of the dual nature of the media as spreaders and correcters of misconceptions. In several crises, such as the Lebanese protests of 2019, economic crisis, the COVID-19 pandemic, and the Beirut Blast of 2020, the function of platforms such as WhatsApp has been critical in the spread of audio misinformation in Lebanon, where emotional voice messages convey false authority, spread outright fear, and sometimes contain actions items to spread the misinformation even further [2]. It can be appreciated in this rush to spread information that media sources are not constrained by peer-reviewed verification of health claims, as balancing speed and accuracy on their part has been a continued challenge in Lebanese media sources, including but not limited to the monitoring of false statements on social media platforms and posting the correct information as verified. For journalists, the infodemic created new challenges: dynamic development of the virus meant that guidelines would change with every passing night, often. Thus, accuracy amid pressures to publish updated information daily became a challenge. Empirical studies in Lebanon during the COVID-19 outbreak have demonstrated that higher trust in media and information sources not only reduces belief in health misinformation but also correlates positively with the adherence to preventive behaviors such as mask-wearing and social distancing [21]. However, the media itself has huge challenges. Profit-making reportage and the lack of specialist science journalism add to it. The lack of official fact-checking, combined with an obligation to drive traffic by sensational headings, has been responsible for the fast spread of false information about health matters. In the absence of legal recourse, there is a non-binding everyday responsibility that is mainly rooted in public indignation rather than binding regulation. This is in accordance with international research that social media sites and news agencies spread health misinformation through algorithms that create feedback loops by rewarding engagement. Around the world, there are governments like Singapore and France that have enacted legislation that holds media organizations accountable for health information dissemination. The Protection of Online Falsehoods and Manipulation Act (POFMA) in Singapore not only has the authority of taking down these pieces of information in hours, while in France, the Fake News Law of 2018 sets a 48-hour period for the removal of misinformation on news organizations and social media platforms [22,23]. The Philippines, on the other hand, has the Bayanihan to Heal as One Act, an act that imposed penalties of jail time or fining people for distributing information about COVID-19 that was not true, leading to the arrest of more than 300 people, an act that poses a risk of violating the right of the public towards critical journalism [24]. Russia has also made the spread of knowingly untrue information about threats to public health and safety an offense with fines and imprisonment of journalists who write about topics related to the COVID-19 pandemic [24]. There are no such laws in the country of Lebanon, and this is another reason that calls for changes in the current system so that fact verification processes are mandatory, along with fact-checking collaborations with qualified medical professionals. For the spread of misinformation related to healthcare to cease while still respecting the freedom of expression, scientists propose strategies like collaboration between the government, media, and the healthcare sector [25].

### Social media and influence on public health

Persons with inadequate access to care and/or inadequate health literacy were considered more likely to turn to social media or alternative, unproven sources. Marginalized groups, such as refugees and migrant workers, in Lebanon have been rendered even more vulnerable to health misinformation with their inadequate access [26]. Various religious and cultural views also appeared to influence views on evidence-based practice with respect to vaccine reception and medical treatment, with health literacy targeted, especially by media and medical leaders, becoming critical in countering the impact of false and/or deceptive claims on health practices. Social media has been the dominant means of carrying medical misinformation in Lebanon. High exposure to unverified news on platforms like WhatsApp and Facebook in Lebanon contributed to widespread conspiracy beliefs, such as the virus being man-made or a tool for population control, which significantly reduced vaccination intent during the infodemic [8]. Algorithmic amplification, viral entries, and health information by non-specialist commentators were given high priority by all groups of respondents. Public health researchers were concerned about the potential of social media to amplify unsubstantiated information, particularly during periods of crisis like COVID-19. The COVID-19 outbreak in Lebanon revealed weaknesses as much as opportunities within the national health communication system of the country. As individuals grew more uncertain, so did the pace at which misleading information circulated, anything from denial of the virus itself to questioning vaccine safety. But things changed as Lebanon’s economic crisis deepened: shortages of medicines and collapsing hospital access systems turned social media into a stage of outrage. Conversely, vaccine hesitancy due to misinformation exhibited repeated global patterns. Indeed, the Covid-19 pandemic marked a turning point in how misinformation spreads because erroneous information regarding treatment, vaccine, and government actions spread faster than mainstream news. This allowed a country like Lebanon to go through the pandemic less adversely than most countries. Cross-national studies confirm that health misinformation is amplified faster than accurate information because algorithms reward “engaging” content [6]. Cross-national analyses further demonstrate that greater engagement with unreliable online content predicts significant increases in infection rates during pandemics, with varying regional impacts across Europe [27]. Their populations are also found to be more likely to be believing and disseminating fake medical information through experiments [28].

This has led to the establishment of policies by nations such as Germany, Singapore, and the UK. In Germany, the Network Enforcement Act (NetzDG) mandates the removal of obviously illicit posts, including misinformation, on social media sites with more than two million registered users in under 24 hours or face heavy penalties [29]. The Protection from Online Falsehoods and Manipulation Act (POFMA) in Singapore in 2019 gives state officials functions to issue correction or delete any false information in public interest, with harsh punishments in case of violation [30]. The UK NHS Misinformation Response Unit, for instance, engages social media platforms directly to tag, correct, and remove hazardous health-related information [31]. There are no such cooperative structures in Lebanon. The Ministry of Public Health has relied primarily on press announcements and alerts, which have not been sufficient in an ever more dynamic digital space. During the COVID-19 pandemic, real-time adjustments on social media were attempted by the Ministry, but these have been reactive rather than pre-emptive in nature. Media professionals recommended that Lebanon initiate partnerships with online platforms to locate misinformation health content and invest in online literacy programs to facilitate the public in distinguishing true from false information.

### Role of health professionals

The medical field has recently shown great importance in the spread of health information misconceptions and correction. Medical professionals have long been viewed as knowledgeable information resources. These professionals have found social media emerging within this context, which creates new dynamics. The Lebanese medical community has shown growing involvement in addressing the misconceptions. The Lebanese medical community showed modest levels of awareness about relatively new medical topics such as the monkeypox outbreak, better attitude levels being associated with increased levels of awareness [32]. Such medical professionals can benefit greatly through specific training to improve the process of addressing health misconceptions. Medical professionals in some countries have used the media to disseminate accurate information when the world encountered the pandemic. Social networks have provided the platform for the rapid sharing of health information, both trustworthy and false. One of the alarming phenomena highlighted by both interviewees is the sharing of false health information that deters medically necessary procedures in favor of alternative treatments that do not work. They still have to contend with various obstacles such as the relatively low popularity of scientific information, lack of institutional trust, and little proficiency in sharing health information via the internet. Healthcare professionals in Lebanon found themselves grappling with the COVID-19 infodemic, with trust-based communication and trustworthiness of sources being factors for the adoption of false news spread on social networks [21]. However, until a fundamental transformation is made in the way in which care is provided and received in this country, professionals in this industry are taking matters in their own hands. This is not an easy job. Scientific information is not as engaging as information that is associated with emotion or entertainment. Professionals in this industry have a great chance to dispel myths and misconceptions that exist within society. However, constraints in terms of time constraints and fear of retaliation soon obstruct their attempts to disseminate information on social platforms [33]. The time has come to abandon traditional approaches to communicating health information. This includes posters and flyers or institutional websites. While this is an effort that deserves credit and was individually effective to a great extent, it lacks a systemic approach. International experiences make it quite clear that outreach is an effort worth engaging in; however, this is unlikely to happen if this is not an institutionally supported process [34]. Initiatives such as the Duke Program on Medical Misinformation include training to enhance clinicians’ abilities in countering patient inquiries about health information and feature adequate approaches to health and evidence-based arguments against such health misinformation [35]. Subsequent to this awakening, other nations such as the UK and other countries around the world now include digital health skills in their medical curriculums to enhance online health advocacy skills in health professionals and individuals alike [36]. Moving forward, as recommended by participants in this research, a combined fight through health professionals and other approaches such as health agencies and health organizations needs to be adopted to tackle health information effectively and effectively uproot health misinformation. Another repeated recommendation was that simplified and scientifically supported access to scientific information, for the general public, could be made more widely available through education. Joint health literacy efforts between healthcare professionals and the media in campaigns for critical thinking have proved successful in enabling communities to effectively distinguish reliable information [37]. These could be in the form of outreach organizations in universities, schools, political parties, or media. Health literacy campaigns ranked quite prominently on the list, enabling people to critically assess medical claims on the internet. Graduate medical education programs around the world encourage residents in these programs to use their expertise for public engagement activities in order to counter misinformation about health issues [38].

### Legal and regulatory challenges

In the context of the European Union, digital media has been subject to regulatory mechanisms in order to combat misinformation through the Audiovisual Media Services Directive (AVMSD) in order to make the spread of original content feasible on digital media platforms [39]. Although the AVMSD was framed as a tool to regulate television broadcast services, it has been modified in the year 2018 to also apply to video-on-demand services and video-sharing websites such as YouTube [40]. The amendments placed advertising content and user content in the regulatory sphere, in effect holding internet media like YouTube and Facebook accountable for carrying false health information. Bans on risky medical advertising that are widespread in conventional broadcasting are now applied to the internet too. Content moderation obligations and age verification obligations also block misinformation regarding health from causing harm without infringing on free speech [41]. Other legislation developed among the frameworks in the EU include Digital Services Act (DSA) in the European Union, 2023, and Strengthened Code of Practice on Disinformation, 2022. These pieces of legislation are unique in that they ensure transparency of fact-checking and the removal of disinformation on large technology companies, such as Google and Meta, with fines of up to 6% of global turnover [42,43]. The French Law 2018–1202 was a great example in the EU where one could destroy false health information instantly and have judicial authorities act efficiently [23]. Both the French and German publics are highly media literacy competent and institutionally confident and were better equipped to suppress health-related disinformation.

In LMICs, quick content deletion and punishments are given importance in acts on misinformation, although they have been a concern for freedom of expression. The Information Technology (Intermediary Guidelines and Digital Media Ethics Code) Rules in the Indian government in 2021, an amended version of the original Act of 2000, mandates the tracing of messages and quick deletion of misinformation, which affects deliberations on censorship and privacy in larger democracies globally [30]. China’s 2016 Cybersecurity Law criminalizes the fabrication and dissemination of fake news that upsets economic or social order. It demands that platforms put in place real-name registration and immediately delete forbidden content, with fines for failure to comply are up to 500,000 yuan [30]. Likewise, Arabic countries like Egypt and the UAE, has taken a proactive approach relating to a cooperative relationship between security forces and media in handling misinformation. The Egyptian Law No. 180 of 2018 on Regulating the Press and Media prohibits publishing false information that threatens national security or disturbs public peace, allowing the Supreme Media Council to suspend or block websites and social accounts with over 5,000 followers that spread fake news inciting violence or hatred [30]. Based on Federal Decree-Law No. 34/2021 in the UAE, spreading false or deceiving information that goes against government-related announcements on health, where there will be fines and jail terms [44]. Also, in a pandemic related to Covid-19, fines amounting to 20,000 dirhams were set regarding spreading incorrect medical information to efficiently reduce misinformation in the UAE [45]. Legislative measures, combined with public education and government trust, have been strong in suppressing health misinformation [15]. Yet, misinformation tends to have its origin in foreign-based websites, which place it beyond national reach. Even for the EU, enforcement of AVMSD rules is limited by legal cross-border restrictions [39].

Lebanon does not have a unified law tackling health-related misinformation. Lebanon still applies its 1943 obsolete media law in the absence of uniform medical misinformation provisions. Liberal media cultures make for extensive content dissemination, even were potentially harmful. Where there is low institutional trust—i.e., in Lebanon—this is even more crucial. However, progress towards more systematic systems continues. Even with all these efforts, Lebanon lacked legal measures of accountability. One legislative tool against health misinformation is long overdue to enable advanced regulation and promote institutional duty. Government agencies, like the Ministry of Public Health (MOPH), play an important part in fighting health misinformation in Lebanon given that there are no observable laws and regulations governing communication related to health. The Ministry of Information’s partnership with WHO, UNICEF, and UNDP during the COVID-19 pandemic exemplifies reliance on collaborative, non-binding initiatives rather than strict legal measures to counter misinformation [46]. Enforcement has proven challenging due to a lack of capacity, sectarianism in media, and confusing government communication [14]. The Health Ministry officials justified that prosecution is not easy because it requires overwhelming evidence of harm and judicial processes are typically slow and ineffective. The burden of proof usually rests with hurt individuals, who must initiate action. It is a costly, time-consuming path seldom pursued except where the Order of Physicians intervenes. Lawsuits on misinformation grounds demand uncomplicated evidence of public damage, something that is hard to prove. Therefore, legal deterrence is also weak, especially in the virtual world. Legal efforts at managing speech quickly run afoul of constitutional constraints, with enforcement being no simpler. A lack of legal deterrence in Lebanon has seen health institutions act only after misinformation has been circulated. Significant lawsuits were never brought against COVID misinformation, particularly by doctors or healthcare institutions. This implies that professional syndicates, especially medical boards, have a greater part to play in regulating misinformation than the courts of law. Such institutions would generally react more swift and sterner than the judiciary. Platform-specific concerns are that the legal context is especially out of date with regards to social media. Traditional older media like television and papers are controlled by 1943 media legislation, but no such law controls online content. A 2018 internet law vaguely refers to social media but is not applied very frequently. Television and the press, in contrast, operate on more established legal foundations—although there, too, accountability is limited. A culturally relevant example showed the problem of unintended misinformation, when two prominent cases of suicides gained much attention, the local media highlighted them as part of a rising trend, while in reality, the nationwide numbers are unchanged. Unsuccessful prosecutions of persons who spread false information in health during the pandemic led to a reluctance in the population to take vaccines and mistrust of the government. In the absence of proper legal enforcement, awareness generation and mobilizing professional syndicates remain more effective techniques. Nevertheless, there is inadequate systemic supervision. Specialists recommend resort over options such as fact-checking software and media education campaigns that prepare individuals to critically evaluate health information, rather than legal repression in a vacuum [41].

### Integration of theoretical frameworks

To further enhance our interpretation of our results, we bring in two existing frameworks that are specifically applicable for infodemics. These are the World Health Organization’s Infodemic Management Framework and the Health Belief Model (HBM). The two organizations listed above offer a structured framework of understanding systemic reactions towards misinformation in general. The World Health Organization’s Infodemic Management Framework has a structured framework of approaching information overwhelm in health crises by ensuring focusing on four key actions: Listening Actively for Concerns, Promoting Understanding for Risks & Expert Views, Enhancing Resilience Against Misinformation, and Engaging for Positive Actions [47]. This framework’s categories fit the descriptions provided by our stakeholders. For example, the role of such sites as fact-checkers, chatbots, and social media monitoring employed by the Ministry of Public Health (MoPH) in connection with COVID-19 relates to “promoting understanding” and “building resilience,” as outlined by Interviewees 6 and 7. But the challenge of inadequate active listening activity, such as when vulnerable sectors have been less engaged because of socioeconomic issues within countries like Lebanon, indicates where the country has not met the standards set through this framework. This framework would be beneficial in shaping the policies of a country, such as through fully establishing a media monitoring unit and providing competency training to health officials, which has already been effective in countries such as Germany and the UK. To complement this systemic perspective, the Health Belief Model (HBM) outlines the influence of six health behavior variables: perceived susceptibility, severity, benefits, barriers, cues to action, and self-efficacy [48]. To inform our study, low health literacy and economic difficulties, described by Interviewees 1, 2, and 5, increase barriers for the verification of information, and the politically charged media Presents deceptive “cues to action” in susceptibility to misinformation, such as anti-vaccine behaviors. The Participants’ Interest in health education relates to the improvement in self-efficacy, which helps improve critical evaluation for sources. The merger of both perspectives suggests the implementation of health literacy for vulnerable groups to modify from Information from UnVerified sources from Socal media platforms. Together, these models emphasize the importance of blending different approaches in LMICs such as in Lebanon: structural change through the WHO framework and behavioral modifications through HBM models. Integrating these different models helps to contextualize our results in addition to making recommendations to policymakers.

## Policy recommendations

To disseminate facts about public health issues in Lebanon, a planned strategy, especially regarding the regulation of healthcare information on television and social media, needs to be adopted. First, setting up a special authority in MoPH can be a very essential first step in tackling the phenomenon of misinformation. It would be very critical to ensure that through a set of strict guidelines among media professionals, public health information on various issues would be factually authentic, scientifically supported, and not based on any form of dramatization. Additionally, creating a “rapid response team” as a quick counteractivity against misinformation would be achieved through a team in the E-Health Program at MoPH, allowing quick correction work through a team of professionals in a 24–48-hour timeline through media postings, based on experiences from COVID-19 efforts and following WHO recommendations. Second, among the recommendations made would be setting up a fact-checking platform to be established as a special authority in MoPH, along with a strategic partnership from “Ministry of Information” and professional “syndicates” like “Lebanese Order of Physicians.” This hub would track health claims in real-time media platforms, leveraging technology to automate the identification of inaccuracy and creating an interim space for public claims validation. The fact-checking technology developed in other nations such as Singapore and the UK, as mentioned in the previous point, are exemplary models to learn from and apply in the same way in the Lebanese system. Thirdly, one of the important challenges in tackling misinformation lies in the weak implementation of regulation policy. Increasing the regulatory policy implementation together with the formulation of new norms in line with the digital era can contribute to the development of a more systematic way of holding individuals accountable.

However, the current formulation of new policies in the regulatory practice must focus on developing amends to the existing 1943 media law through the incorporation of an exemplary task force made of representatives from the MoPH, the Ministries of Justice, and legal advisors in no more than 12 months, with the emphasis placed on defining medical misinformation based on the thresholds of potential harm in the first application of such models in the Lebanese system. An important aspect in this regard would be the interaction of the media and the medical sector in fighting misinformation. Media and social media influencers need workshops and training programs on how to deal with health misinformation and promote accurate sources of health. In this regard, mandatory health communication training would be important for all medical journalists, with certification from the Ministry of Information on an annual basis. Engaging the public in programs and campaigns on critical thinking and verification of sources could be crucial in helping people deal with health information in the correct manner. Encouraging relationships between medical institutions and the media would also be important in raising public confidence in medical advice and guidance. The integration of digital health communication in medical programs, as seen in the UK example, would be important in providing medical doctors with the requisite skills in dealing with health misinformation online. However, the root of the problem also has to do with the issue of health literacy, which plays an important role in defining how the information given to the public in medical fields is responded to. So, in the quest to make the issue of health literacy better, the government needs to invest in education, from incorporating critical health education in schools to running campaigns in the country. Socioeconomic factors can also not be ignored since, in some communities with less access to healthcare and education, the levels of susceptibility to information can be high.

## Limitations

Several limitations should be considered while interpreting the outcomes of this study. First, despite efforts to survey a variety of stakeholders, access was limited to a few key players, most significantly legal commentators, policymakers, and media representatives. Our sample focused on elite actors, excluding perspectives of patients, grassroots members, nurses, or mid-level media staff, which may limit representation of lived experiences. Second, qualitative design limits generalizability. Though the in-depth interviews yielded rich contextual insight, absence of data triangulation in the form of legal case study, enforcement statistics, or media content analysis limits the scope of findings. Third, social desirability bias may affect the responses given by the respondents, particularly those from regulatory institutions or social media platforms. Responses may also have indicated closer alignment with official pronouncements rather than actual problems, overstating the effectiveness of existing measures to limit misinformation. Fourth, the fast-changing environment of digital misinformation policy is a challenge to capturing the full breadth of regulatory interventions. Longitudinal methods in future research would be a welcome development to follow changes in legal frameworks and enforcement patterns over time. Finally, access to certain legal documents, policy discussions, and enforcement materials was limited. Although publicly available law and policy declarations were analyzed, interagency deliberations in government or private stakeholders were not available. Future studies might be assisted by improved access to legislative drafts, enforcement data, and judicial rulings. In addition, future studies should incorporate viewpoints from diverse social media users, patients, lay people, and nurses, who are key in health information dissemination.

## Conclusion

Preventing public health misinformation in Lebanon requires an institutional, synchronized, and anticipatory approach. This approach should be facilitated by good political will and institutional capacity that facilitates collaboration between policymakers, media practitioners, and healthcare workers. Another key to these solutions is health literacy. Enhancing health literacy is central to how individuals interpret and respond to medical information. Ultimately, prioritizing health literacy in national strategy will enable Lebanon to build a stronger society where citizens are knowledgeable about their health and better protected from misinformation. All in all, this study provides useful insights into the regulation challenges of health misinformation in Lebanon and highlights important areas where policies must be formulated. It is also a reference for other LMICs that are facing comparable challenges in regulating digital health misinformation.

## Supporting information

S1 File Full interview guide used for semi-structured interviews. (DOCX)

S2 File Course syllabus for the Social and Preventive Medicine course, which includes the IRB exemption details. (DOCX)

S1 Table Codebook for thematic analysis. (DOCX)
