# Supplementary material for: Medical misinformation in Lebanese media: A qualitative study of Stakeholders’ perspectives and policy gaps
Source: PLOS Glob Public Health. 2026 Apr 8;6(4):e0006277. doi: 10.1371/journal.pgph.0006277 (PMC13061186; doi:10.1371/journal.pgph.0006277)
Supplement: S1 File — (DOCX) [file pgph.0006277.s001.docx]

**S1 File: Question Guide**

1. Can you describe your role in combating health misinformation and ensuring the dissemination of accurate medical information to the public?

2. What criteria do you use to identify trustworthy sources before engaging in discussions about healthcare?

3. What responsibilities do you assume when addressing the spread of misinformation during pandemics like COVID-19? Could you share your experiences related to COVID- 19?

4. How can we address the issue of sociodemographic factors influencing the reception of misleading information in Lebanon, especially among communities with limited access to proper healthcare resources? What approaches should be taken to tailor health messages for diverse communities?

5. How can stakeholders such as the media, healthcare providers, and the government collaborate to enhance public trust in health information?

6. What are the primary factors that contribute to the spread of health misinformation in both online and traditional media within Lebanon? Based on your experience, what impact does misinformation about medical issues have on public health behaviors?

7. How can editors balance the need for rapid reporting with ensuring accuracy, especially concerning health-related news? How do you handle the challenge of simplifying complex medical topics without oversimplifying or distorting key information?

8.

At what point do attention-grabbing phrases become misleading, and how do you determine where to draw the line?

9. What potential do you see for artificial intelligence in fact-checking, particularly when timely reporting is essential?

10. What steps do you take when incorrect or misleading health information is published? What about addressing misinformation when it originates from widely followed public figures or influencers?

ھل یمكنك وصف دورك في مكافحة المعلومات الصحیة المضللة وضمان نشر المعلومات الطبیة الدقیقة للجمھور؟ 1.

ما المعاییر التي تستخدمھا لتحدید المصادر الموثوقة قبل الانخراط في مناقشات حول الرعایة الصحیة؟ 2.

ما المسؤولیات التي تتحملھا عند التصدي لانتشار المعلومات المضللة خلال الأوبئة مثل كوفید 19- ؟ ھل یمكنك 3. مشاركة تجاربك

المتعلقة بجائحة كوفید 19- ؟

كیف یمكننا معالجة مسألة تأثیر العوامل الاجتماعیة والدیموغرافیة على استقبال المعلومات المضللة في لبنان، خاصةً 4. بین

المجتمعات التي تعاني من محدودیة الوصول إلى الموارد الصحیة المناسبة؟ ما الأسالیب التي ینبغي اعتمادھ ا

لتكییف الرسائل الصحیة مع المجتمعات المتنوعة؟ كیف یمكن لأصحاب المصلحة مثل وسائل الإعلام ومقدمي الرعایة

الصحیة والحكومة التعاون لتعزیز ثقة الجمھور 5.

بالمعلومات الصحیة ؟

ما العوامل الرئیسیة التي تسھم في انتشار المعلومات الصحیة المضللة في وسائل الإعلام التقلیدیة وعبر الإنترنت في 6.

لبنان؟ ومن خلال خبرتك، ما التأثیر الذي تتركھ المعلومات المضللة حول القضایا الطبیة على سلوكیات الصح ة

العامة؟ كیف یمكن للمحررین الموازنة بین الحاجة إلى سرعة النشر وضمان الدقة، خاصةً فیما یتعلق بالأخبار الصحیة؟

كیف 7.

تتعامل مع تحدي تبسیط المواضیع الطبیة المعقدة دون الإفراط في التبسیط أو تشویھ المعلومات الأساسیة؟

في أي نقطة تصبح العبارات الجذابة مضللة، وكیف تحدد الخط الفاصل في ھذا السیاق؟ 8.

ما الإمكانات التي تراھا للذكاء الاصطناعي في التحقق من الحقائق، خاصةً عندما یكون من الضروري الإبلاغ في 9. الوقت

المناسب؟

ما الخطوات التي تتخذھا عند نشر معلومات صحیة غیر صحیحة أو مضللة؟ وماذا عن التصدي للمعلومات المضللة 10.

عندما یكون مصدرھا شخصیات عامة أو مؤثرین یحظون بمتابعة واسعة ؟__
