# Supplementary material for: Medical misinformation in Lebanese media: A qualitative study of Stakeholders’ perspectives and policy gaps
Source: PLOS Glob Public Health. 2026 Apr 8;6(4):e0006277. doi: 10.1371/journal.pgph.0006277 (PMC13061186; doi:10.1371/journal.pgph.0006277)
Supplement: S2 File — (DOCX) [file pgph.0006277.s002.docx]

**S2 File: Course Syllabus**

**Practicum in Social and Preventive Medicine**

**(IDTH 268- SPMIV)**

**2024-25**

**Course Coordinators:**

Salim M. Adib, MD, DrPH

Office: Van Dyck room 221

Ext: 4640

Email: **sa193@aub.edu.lb**

**Introduction**

During their four years in medical school, students have the chance to acquire important medical and technical skills and to learn a great deal about health in general. Over the four years students are also exposed to different courses in public health that describe the relationship between medicine and public health. In this short but intensive clerkship, we hope to build on the students’ accumulated knowledge and experiences in public health. The course has 2 components: a research project and discussion sessions.

- The **research project** requires students to investigate the social and preventive aspect of a selected medical problem. This will be done through reviews of the literature, desk review of data and interviews with stakeholders.
- The **discussion sessions** with relevant experts focus on the Lebanese health system as well as on the interaction of the disciplines of medicine and public health.

**Learning outcomes**

By the end of this clerkship, students will be able to:

1. Critically appraise the social health dimensions of a health problem.
2. Understand the challenges of addressing those dimensions within the local Lebanese context.
3. Know the basic principles of qualitative research.
4. Appreciate the leading advocacy role that physicians can play on behalf of their communities.

**Readings**

1. Primary Health Care Systems in Lebanon (2017). El-Jardali et al. https://www.who.int/alliance-hpsr/projects/AHPSR-PRIMASYS-Lebanon-comprehensive.pdf?ua=a1
2. Rapid Appraisal Concepts and Techniques. Manual by Robin Heath.

**Course Requirements and Grading**

At the end of the clerkship, students will orally present their project to faculty and students. Within 48 hours from the end of the clerkship, the students must submit a soft copy of their final report to their advisor and course coordinator. The discussion sessions will be scheduled at the beginning of the rotation. Students are expected to read all assigned material prior to the sessions and are encouraged to actively contribute to the discussion of the readings.

The final grade will be a composite of a group grade (based on the written report and presentation of the project), and an individual grade (based on contribution to the team project). Grading will be as follows:

**Activity % of Grade Description**

**Written Report** 40% Group grade

**Presentation 25% Group grade**

**Advisor’s evaluation* 10% Individual grade**

**Peers’ evaluation* 20% Individual grade**

**Coordinator’s evaluation** 5% Group grade

* Reflects your contribution to the discussions, the project implementation, the quality of the work presentation, and your ability to work in a team.

**Research Ethics and IRB**

We have obtained an exemption from the IRB for all projects related to this clerkship. This is based on the assumption that all projects would meet the following criteria:

- The interviewees are leaders in their field
- Oral informed consent is being sought from all interviewees (no written consent form is required, no signature required )
- If interviewees mention any sensitive information, the students are instructed to disregard that information and not include it in the presentation or the report.

**Final Report**

The report should not be longer than 3,000 words. The cover sheet should include the title of the paper, the names of all group members, the name of the group advisor and the month/year of the clerkship.

The preferred structure is as follows: Introduction, Methods, Results, Discussion, and References.

- The introduction should include a brief introductory statement of the problem and its significance (why is it interesting or important to research?) e.g. the problem is new, or widespread, or has a high burden of disease, or is increasing, or changing etc.
- The literature review should be structured to support the rationale of your study (supportive evidence of why your problem is interesting, and why is it important to research it the way that you intend to, i.e. why your study is important). This part ends with your research question and the objectives of the study that aim to answer the question.
- The methodology section should describe the qualifications of the sources of data and the settings of the interviews, as well as the qualitative analysis procedures to be used.
- The results should include all the pertinent findings from your research.
- The discussion includes study limitations and your reflections on the results.
- The conclusion includes recommendations for policy, practice or future research.
- References can be in any of the standard accepted styles as long as they are complete and consistent.

**On proper referencing and plagiarism**

Education is demanding and you need to properly manage your time. Do not hesitate to use the resources around you but do not cut corners. Cheating and plagiarism will not be tolerated. Review the Student Code of Conduct and familiarize yourself with definitions and penalties (p. 142-144 of the Student Handbook, 2003-2004). If you are in doubt about what constitutes plagiarism, ask your instructor because it is *your* responsibility to know. Remember that the American University of Beirut has a strict anti-plagiarism policy. Do not become a lesson to others. The use of IT sources is allowed, providing this is clearly specified in “source of data” and in the references. Full-fledged “cut and paste” of an IT document is discouraged and will result in the downgrading of the report’s assessment.
