# Supplementary material for: Medical misinformation in Lebanese media: A qualitative study of Stakeholders’ perspectives and policy gaps
Source: PLOS Glob Public Health. 2026 Apr 8;6(4):e0006277. doi: 10.1371/journal.pgph.0006277 (PMC13061186; doi:10.1371/journal.pgph.0006277)
Supplement: S1 Table — (DOCX) [file pgph.0006277.s003.docx]

**S1 Table: Codebook for thematic analysis**

| **Main Theme** | **Sub-Code** | **Definition** | **Example quote** | **Notes** |
| --- | --- | --- | --- | --- |
| Societal & Information Environment | Health literacy gaps | Low public education / limited science literacy that reduces ability to evaluate health claims. | “There's a lack of awareness and education around scientific and health issues in Lebanon… health and science journalism are not well developed in this country.” (Interviewee 2).  PH project resubmission final | Central vulnerability across interviews; links to simplification challenges in media. |
| Societal & Information Environment | Economic hardship | Financial barriers that push people to online/unregulated sources instead of clinical care. | “The ongoing economic crisis has rendered healthcare less accessible, pushing people to the internet for explanations… Many people cannot afford a hospital visit, so they resort to the internet.” (Interviewee 4).  PH project resubmission final | Tied to access & digital divide; increases susceptibility. |
| Societal & Information Environment | Cultural / religious beliefs | Preexisting cultural or religious worldviews that make some claims more believable. | “People are likely to believe information that is consistent with their religious or cultural beliefs.” (Interviewee 6).  PH project resubmission final | Emergent code linked to vaccine hesitancy examples. |
| Societal & Information Environment | Language / translation barriers | Poor translation or language complexity that distorts medical content. | “Occasionally poorly translated information will be misread and relayed incorrectly.” (Interviewee 6).  PH project resubmission final | Important for immigrant / low-literacy groups; affects accuracy of international sources. |
| Media Outlets' Role | Sensationalism & speed | Prioritizing fast/engaging stories that outpace fact-checking and spread misinformation. | “One of the greatest challenges is the speed at which misinformation gets around, and it tends to get around quicker than individuals who are trying to debunk it.” (Interviewee 1).  PH project resubmission final | Linked to social media algorithms and “first-heard” bias. |
| Media Outlets' Role | Fact-checking practices | Voluntary editorial processes to verify health claims before publication. | “We always refer to medical professionals to fact-check health claims before we go on air. We also have a specific TV segment for dispelling misinformation.” (Interviewee 1).  PH project resubmission final | Present in some outlets (MTV, Al-Nahar) but not uniformly enforced. |
| Media Outlets' Role | Simplification challenges | Difficulty balancing accessible language with technical accuracy. | “We… take many courses… to master the art of simplification… our message… should be directed to the population in general.” (Interviewee 1).  PH project resubmission final | Ties to health literacy gaps; training a mitigation strategy. |
| Media Outlets' Role | Inadvertent errors (case examples) | Unintentional misinformation arising from haste, poor context, or misframing. | “Our segment attempted to introduce potential side effects... It was never our aim to panic or lead patients to stop medication.” (Interviewee 1) — beta-blocker (Concor) example.  PH project resubmission final | Distinguish from deliberate disinformation; useful for training and corrections. |
| Health-Care Sector Involvement | Clinician as influencer | Clinicians using social media / media platforms to disseminate or correct health information. | “I see it as a big responsibility to share accurate health information with the public. My goal is to empower patients with knowledge.” (Interviewee 3).  PH project resubmission final | Dual role: authoritative voice but constrained by time, reach, platform skills. |
| Health-Care Sector Involvement | Patient self-diagnosis risks | Harm from patients acting on online misinformation (self-treatment / delayed care). | “The reverse self-diagnosis using fallacious content can be dangerous.” (Interviewee 4). | Clinical consequence; links to unsafe alternative treatments. |
| Health-Care Sector Involvement | Industry influence / fear marketing | Commercial actors use fear or misleading claims to sell services/products. | “A lot of times, it is due to companies wanting to sell IVF or other drugs.” (Interviewee 3). | Important to flag in policy recommendations (advertising regulation). |
| Health-Care Sector Involvement | Engagement strategies (storytelling) | Using narratives and tailored content to increase reach of evidence-based messages. | “I like to employ more stories, which is one of the most powerful ways to engage people.” (Interviewee 3). | Practical mitigation combine evidence with storytelling. |
| Government Interference / Role | Crisis communication | Government briefings / official channels used to calm, inform, and lead during crises. | “The last thing you want is panic.” (Interviewee 5). | Positive example during COVID; credibility matters for countering misinformation. |
| Government Interference  / Role | Reactivity & fragmentation | Official efforts that are helpful but reactive, uncoordinated, or short-lived. | “What we do is monitor the media and respond with clarifying statements.” (Interviewee 7). | Need for proactive, sustained strategies (recommendation gap). |
| Government Interference  / Role | Investment in health literacy | Long-term public education to build resilience against misinformation. | “The more you invest in health literacy and public awareness, the harder it is for misinformation to take hold.” (Interviewee 5). | Cross-sector campaigns (schools, media, health) recommended. |
| Government Interference  / Role | Algorithmic / platform challenges | Difficulty amplifying official messages due to social platform algorithms. | “Social media is not level. Algorithms determine what gets amplified.” (Interviewee 5). | Suggests need for partnerships with platforms and platform-specific tactics. |
| Current Legislation & Regulation | Lack of specific laws | Absence of clear, enforceable legal framework targeting online health misinformation. | “There are no specific regulations per se.” (Interviewee 7). | Legal vacuum leads to reliance on 1943 media law and ad hoc responses. |
| Current Legislation & Regulation | Prosecution & enforcement barriers | High burden of proof, slow courts, and rare successful litigation against misinformation. | “Prosecution only if harm is proven… A case that resolves in less than five years is optimistic.” (Interviewee 9). | Points to why syndicates (Order of Physicians) are seen as an alternative enforcement route. |
| Current Legislation & Regulation | Professional syndicates' role | Medical boards/syndicates as quicker ethical-disciplinary bodies to act on claims. | “The Order must be in a position to undertake formal investigations against public health claims.” (Interviewee 4). | Operational lever for accountability when courts are impractical. |
| Current Legislation & Regulation | Outdated frameworks | Reliance on old statutes not fit for regulating digital media. | “Internet law remains vague. We don't rely on it and fall back on ancient 1943 media statutes.” (Interviewee 9). | Clear recommendation area: update laws to the digital era. |
| Lessons from COVID-19 infodemic | Rapid misinformation circulation | Fast spread of false claims under uncertainty (denial, vaccine side-effects, conspiracies). | “Early COVID, we knew very little... Some even claimed the virus never existed.” (Interviewee 7). | Empirical exemplar showing harm and need for monitoring units. |
| Lessons from COVID-19 infodemic | Effective gov't strategies (mitigation) | Tools used: fact-check sites, daily briefings, chatbots, media monitoring. | “Fact Check served as a public window… The chatbot provided quick, accurate responses.” (Interviewee 7 & 6). | These are models to scale/ institutionalize. |
| Prospective Plans / Recommendations | Cross-sector collaboration | Partnership across MoPH, Ministry of Information, media, syndicates for coordinated response. | “The Ministry of Public Health, Ministry of Information... must work together.” (Interviewee 4). | Repeated recommendation, basis for a national rapid-response hub. |
| Prospective Plans / Recommendations | Health literacy campaigns | Preventive education to teach source evaluation and critical thinking. | “We must teach people how to identify credible sources, and outline how misinformation can be more destructive.” (Interviewee 4). | Should be intersectoral (schools, media, clinics). |
| Prospective Plans / Recommendations | Media training & platform partnerships | Training for health journalists; agreements with platforms for moderation and rapid correction. | “Health reporting in Lebanon is underdeveloped... We need direct communication between scientific institutions and the public.” (Interviewee 2 & 4). | Practical items: certification, platform MOUs, funded fact-check units. |
